# Summarizing Specific Profiles in Illumina Sequencing from Whole-Genome Amplified DNA

**DOI:** 10.1093/dnares/dst054

**Published:** 2013-12-18

**Authors:** Isheng J. Tsai, Martin Hunt, Nancy Holroyd, Thomas Huckvale, Matthew Berriman, Taisei Kikuchi

**Affiliations:** 1 Parasite Genomics, Wellcome Trust Sanger Institute, Wellcome Trust Genome Campus, Cambridge CB10 1SA, UK; 2 Faculty of Medicine, Division of Parasitology, Department of Infectious Disease, University of Miyazaki, Miyazaki 889-1692, Japan

**Keywords:** whole-genome amplification, Illumina, SNPs, genome assembly, chimeric DNA

## Abstract

Advances in both high-throughput sequencing and whole-genome amplification (WGA) protocols have allowed genomes to be sequenced from femtograms of DNA, for example from individual cells or from precious clinical and archived samples. Using the highly curated *Caenorhabditis elegans* genome as a reference, we have sequenced and identified errors and biases associated with Illumina library construction, library insert size, different WGA methods and genome features such as GC bias and simple repeat content. Detailed analysis of the reads from amplified libraries revealed characteristics suggesting that majority of amplified fragment ends are identical but inverted versions of each other. Read coverage in amplified libraries is correlated with both tandem and inverted repeat content, while GC content only influences sequencing in long-insert libraries. Nevertheless, single nucleotide polymorphism (SNP) calls and assembly metrics from reads in amplified libraries show comparable results with unamplified libraries. To utilize the full potential of WGA to reveal the real biological interest, this article highlights the importance of recognizing additional sources of errors from amplified sequence reads and discusses the potential implications in downstream analyses.

## 1. Introduction

The use of genomic data generated by so-called ‘next generation sequencing’ (NGS) has become commonplace in many fields of biological research, with sequencing-by-synthesis from Illumina currently the most popular. A standard Illumina paired-end (PE) library is made from DNA templates of about 500 bp in length, and a sequencing run can generate billions of paired reads of length 37–250 bp from either ends of these fragments.^1^ Reads from longer fragments of DNA can also be produced to aid the deconvolution of repetitive regions and for identifying large structural variations in genomes. A specialized mate-pair (MP) library, constructed by introducing a circularization step at the start of the library preparation, allows end sequencing from fragments of at least 2 kb.^2–4^ This powerful technology can be applied to address a wide range of biological questions, such as variant calling and resolving haplotypes between individuals of a population or *de novo* assembly of complex genomes.

Advancement in library preparation also permits their creation from just a few nanograms of DNA.^5^ Nevertheless, obtaining even nanograms of starting material can be challenging in certain applications. One solution is to pool many samples to obtain sufficient DNA to construct one library. However, this approach is often not applicable to rare clinical or archived samples,^6^ and increases the complexity of downstream analysis. Within an assembly of pooled DNA samples, it can be particularly challenging to distinguish variants of a sequence that is repeated in the genome of one individual from allelic differences between multiple individuals. This problem increases with the levels of intraspecies variation, for instance, *C. brenneri* has 14.1% of polymorphic synonymous sites between individuals, comparable with hyperdiverse bacteria.^7^ A potential solution is to use whole-genome amplification (WGA) techniques to reduce the amount of DNA required to make a sequencing library. Several WGA protocols have been proposed^8^ and can be divided into those based on polymerase change reaction (PCR) or those involving multiple strand displacement amplification (MDA). PCR-based techniques such as degenerate oligonucleotide primed PCR^9^ and primer extension PCR^10^ can produce non-specific amplification artefacts and typically short-amplification products.^11^ MDA uses the strand-displacing DNA polymerase from Phi29 bacteriophage and has several advantages over PCR-based protocols. For example, MDA can generate long-amplified fragments up to 100 kb, which are of feasible size to serve as templates from which MP libraries can be synthesized.^8^ An alternative method of amplification called pWGA (primase-based WGA)^12^ is based on *in vitro* reconstruction of the naturally existing cellular DNA replication machinery found in bacteriophage T7, and comparable performance with MDA in terms of efficiency and unbiased amplification has been reported.^12^ WGA techniques have already been regularly used in genotyping bacteria,^13^ other small organisms^14^ and human cells.^15^ Though still in its infancy, sequencing from single cells is now feasible using WGA and this opens up a new field of exploring heterogeneity within populations, or populations of cells in an organism or tissue, an ultimate goal for many geneticists and molecular biologists.

Despite the advances in WGA methods, preferential amplification of certain DNA fragments still exists,^16^ resulting in uneven sequencing between different parts of a given genome and chimera formation.^17^ In this article we describe the effect of amplifying nanograms of genomic DNA, which is usually the available starting material of a *de novo* small eukaryote genome project, with three different protocols before constructing Illumina short- and long-insert libraries. Using the high quality genome sequence of *C. elegans* as a reference, we show that GC content and composition of inverted and tandem repeats play a major role in the variation of read coverage. We demonstrate that reads sequenced from amplified DNA can generate *de novo* assemblies that are of comparable quality with those from unamplified DNA. In addition, we have also examined capillary reads from libraries cloned from amplified high molecular weight DNA of the potato cyst nematode *Globodera pallida* genome project. As the research focus is turning to more unexplored organisms and single cells, WGA protocols will become the standard method of choice. Understanding the caveats and biases generated with these protocols is a paramount requirement for interpretation of results.

## 2. Materials and methods

### 2.1. Whole-genome amplification

Genomic DNA was extracted from ∼10 000 *C. elegans* N2 nematodes using Genomic tip G20 (Qiagen) according to the manufacturer’s instructions. Ten nanograms of template DNA was used in each of three WGA reactions using: (i) GenomiPhi v2 (GE life science), hereafter termed Phi; (ii) Phi29 MDA plus trehalose, hereafter termed Tre; and (iii) Rapisome (BioHelix), hereafter termed Rap. The Phi WGA reactions were carried out in the 20 µl reaction mixture at 30°C for 90 min followed by heat denaturation at 95°C for 3 min according to the manufacturer's instructions. For Rap, DNA was mixed directly with the 25 µl reaction mixture and incubated at 37°C for 60 min according to the manufacturer's instructions. For Tre, Phi29 polymerase (Qiagen), 0.7 M trehalose and 16 h reaction time were used according to the method described in Pan *et al.*^18^ Amplified products were purified using a QIAAmp DNA mini kit (Qiagen) and DNA concentrations were measured using Qubit (Life technologies).

### 2.2. Illumina library construction and sequencing

One microgram of DNA was used to construct standard 450 bp libraries using a TruSeq DNA Sample Preparation Kit with the standard protocol (Illumina), after fragmentation on the Covaris, 3 kb mate-pair libraries were constructed following the protocol described in Park *et al.*^19^ with the following exceptions. Size selection was carried out with Agencourt AMPure XP beads (Beckman Coulter) in a buffer of 5% PEG and 0.95 M NaCl, aiming to remove most fragments of length <1.5 kb. Nick translation was carried out for 11 min (libraries in Replicate 1) or 14 min (libraries in Replicate 2). Libraries were sequenced using the TruSeq SBS Kit v3-HS kit according to the manufacturer's recommended protocol (https://icom.illumina.com/) in either Illumina MiSeq 150 cycles (for 3 kb MP libraries in Replicate 2) or the Illumina HiSeq 100 cycles (for all other libraries). *In situ*, the linearization, blocking and hybridization step was repeated to regenerate clusters, release the second strand for sequencing and to hybridize the R2 sequencing primer followed by another 100 or 150 cycles of sequencing to produce PE reads.

### 2.3. Data analysis

Reads were trimmed based on base quality (an average phred score of 15 for every four bases) and the presence of adaptor sequences. For 450 bp fragment short-insert libraries Trimmomatic^20^ was used with options: ‘*ILLUMINACLIP 2:40:15 LEADING:3 TRAILING:3 SLIDINGWINDOW:4:15 MINLEN:36*’. For 3 kb fragment long-insert libraries an in-house developed algorithm was used to trim biotin adaptor sequence which may present at either ends of the long-insert fragments (Supplementary Fig. S1). Three to 15% of reads were removed in short- and long-insert libraries, respectively, before undertaking the alignment stage (Supplementary Table S1).

Illumina reads were aligned to the *C. elegans* reference genome (WS236 from Wormbase ftp://ftp.wormbase.org/) using SMALT (http://www.sanger.ac.uk/resources/software/smalt/). Repetitive mappings were allowed, and only alignments with at least 80% or 50% of the sequence read aligned to reference were considered in the short- and long-insert libraries, respectively. Additionally, a maximum of 600 bp and 100 000 bp insert size were considered for paired mappings in the short- and long-insert libraries, respectively. Duplicates were called using GATK.^21^ SNPs were called using Varscan2^22^ with options ‘−min-coverage 5—strand-filter 1’ using alignment files (in the form of bam format) as the input on each of the short-insert replicates. Tandem and inverted repeat content of the *C. elegans* genome was calculated using program trf^23^ and irf^24^ with default parameters, respectively. GC content and repeat content analyses for 10 kb windows in the *C. elegans* genome was calculated using a combination of BEDTools^25^ and custom Perl and R^26^ scripts. Capillary reads from the *G. pallida* genome project were first trimmed to increase bases having at least phred quality score of 40 and the vector contamination was removed. The resulting subset of reads was mapped against the *G. pallida* v1 assembly (ftp://ftp.sanger.ac.uk/pub/pathogens/Globodera/pallida/) using SSAHA2.^27^

Assemblies were constructed from short-insert libraries using SGA v.0.10.9^28^ with authors' recommended parameters (https://github.com/jts/sga/blob/master/src/examples/sga-celegans.sh). Scaffolding was performed using SSPACE basic version 2.0^29^ with default settings, with library sizes set to the median insert values as shown in Table 1 with a standard deviation of 0.5. These assemblies were then compared with the *C. elegans* genome (WS236) using GAGE.^30^ Because of the differences in number of reads between replicates, we only presented Replicate 1, which has more sequencing depth (Table 4). A separate Supplementary Table S2 summarizes assemblies where every library is normalized to the library with least number of reads.

**Table 1.** Mapping statistics of sequenced reads from unamplified and amplified libraries

Method	Replicate	Platform	Library type	Total reads	Mapped (%)	Duplicates^a^ (%)	Proper^b^ (%)	Both mapped (%)	Median insert (bp)	Median coverage
Unamplified	1	HiSeq	Short	41 688 676	99.7	0.8	98.1	99.5	285	38
	2	HiSeq	Short	16 333 732	89.4	0.6	88	89.3	357	13
Phi	1	HiSeq	Short	48 501 916	99.4	0.7	98	98.8	224	46
	2	HiSeq	Short	20 656 296	95.2	0.8	88.6	93.7	349	18
Tre	1	HiSeq	Short	44 481 270	99.3	1.1	97.6	98.7	237	40
	2	HiSeq	Short	25 188 788	96.2	0.7	92	95.3	328	22
Rap	1	HiSeq	Short	26 277 398	89.3	2.8	71.6	80.1	248	15
	2	HiSeq	Short	22 278 134	82.5	35.7	60	73.7	308	3
Unamplified	1	HiSeq	Long	60 856 860	99.5	9	97.2	99.1	2631	41
	2	MiSeq	Long	2 551 720	86.4	1	79.1	85.4	2136	2
Phi	1	HiSeq	Long	61 735 210	99.5	3.5	81.5	99.2	2576	39
	2	MiSeq	Long	2 760 576	99.3	1.2	80.2	98.9	2025	2
Tre	1	HiSeq	Long	55 842 586	99.4	3.4	82.6	99	2285	29
	2	MiSeq	Long	2 999 856	99.4	1	73	98.9	2094	2
Rap	1	HiSeq	Long	58 443 656	99.4	7.6	92.1	99	2591	35
	2	MiSeq	Long	3 914 622	99	6.6	91.8	98.7	2121	2

All percentages are relative to total number of reads in each replicate.

^a^Reads that are identical copies of other reads and have exact mapped coordinates on the genome.

^b^Reads mapped in the correct orientation and at a distance corresponding to that predicted by the fragment library size.

## 3. Results

### 3.1. Genome amplification

*Caenorhabditis elegans* genomic DNA was amplified using three different protocols that utilized Phi29 polymerase alone (Phi); Phi29 polymerase supplemented with trehalose (Tre), which reportedly produces a more homogenous and unbiased amplification^18^; and Rapisome (Rap)—a commercially available version of pWGA, which does not utilize random primers and initial template denaturation that are possible factors in chimera formation in MDA reactions. After amplification, we observed DNA fragments of length>10 kb resulting from all amplification methods. The average yield of amplified DNA from 10 ng template DNA was 46 ng/µl of reaction mixture (Phi), 31 ng/µl (Rap) and 95 ng/µl (Tre). These fragments were further fragmented and size selected to generate Illumina libraries (Supplementary Fig. S1).

### 3.2. Illumina read quality

A total of ∼495 million of both standard 450 bp PE (short-insert libraries) and 3 kb MP (long-insert libraries) Illumina reads were generated from amplified *C. elegans* genomic DNA fragments. Two technical replicates were generated from each amplification protocol and unamplified DNA. The availability of a high quality *C. elegans* reference genome allowed the quality of the libraries to be assessed by checking various mapping metrics such as number of reads mapped, orientation of mates in a pair. In all cases, at least 82% of the reads were aligned to the reference genome (Table 1) and the insert size distribution, i.e. the distance between two mates of a read pair (including themselves) mapped to the genome in the correct orientation, was close to our expectation for selecting desired size fragments (Supplementary Fig. S2).

Various errors can occur at different stages of Illumina library preparation (Supplementary Fig. S1). In both short- and long-insert library construction, duplicates can arise during PCR amplification, resulting in perfect copies of the original DNA templates being sequenced many times.^31^ Of the mapped reads in short-insert libraries, the number of PCR duplicates was ∼1%, except in Rap libraries, where Replicate 1 had 2.8% duplicates and Replicate 2 had 35.7% duplicates suggesting that the starting amplified products had lower molecular sizes with only a fraction of DNA desirable as DNA templates. We therefore considered Replicate 2 as a ‘failed’ library and excluded it from subsequent analysis. The number of PCR duplicates in long-insert libraries was consistently higher than in short-insert libraries, as observed by others.^4^ The number of PCR duplicates also appeared to be affected by individual runs; a consistently fewer duplicates were observed in the second replicate. Again, we found that PCR duplicates in Rap amplified long-insert libraries were high in both replicates.

Problematic reads can arise from several stages of long-insert library construction. First, fragments can be produced from circularized templates where nick translation has occurred in a segment that does not contain a biotinylated adaptor (Supplementary Fig. S1) presumably the selection stages are sometimes ineffective. We found out that 0.5–4.8% of such reads present in long-insert libraries and they appear to be higher in Replicate 2 and not influenced by WGA protocols (Table 2). Second, templates can still be circularized if size selection is not efficient (Supplementary Fig. S1). Paired reads sequenced from these fragments should have short-insert sizes and be aligned in the correct (outward-facing in long-insert libraries) orientation. We found these reads to be present at much higher frequency in Replicate 2 which were sequenced as a batch (Supplementary Fig. S2).

**Table 2.** Mapping statistics of improperly paired sequenced reads from unamplified and amplified libraries

Method	Replicate	Library type	Singletons (%)	Interchromosomal^a^ (%)	Outies/innies^b^ (%)	Wrong orientation^c^ (%)	Incorrect insert size (%)
Unamplified	1	Short	0.2	0.5	0.5	0.2	0.2
	2	Short	0.2	0.6	0.3	0.1	0.3
Phi	1	Short	0.6	0.15	0.1	0.55	0.0
	2	Short	1.5	0.4	0.3	4.1	0.3
Tre	1	Short	0.6	0.2	0.1	0.8	0.0
	2	Short	1	0.4	0.3	2.5	0.1
Rap	1	Short	9.1	4.2	0.3	3.7	0.3
	2	Short	8.8	0.5	1.7	3.1	8.4
Unamplified	1	Long	0.4	1	0.5	0.3	0.1
	2	Long	1	3.9	1.4	0.8	0.2
Phi	1	Long	0.3	2	1.7	13.3	0.7
	2	Long	0.4	5.1	3.1	10.0	0.5
Tre	1	Long	0.4	1.9	1.0	12.9	0.6
	2	Long	0.4	8.1	4.8	12.4	0.6
Rap	1	Long	0.4	1.3	0.5	4.5	0.6
	2	Long	0.3	3	1.3	2.2	0.4

All percentages are relative to total number of reads in each replicate shown in Table 1.

^a^Reads with mates mapped to different chromosomes.

^b^Reads with mates mapped to the same chromosome that show incorrect orientation of facing either outwards (‘←→’; outies for short-insert libraries) or inwards (‘→←’ innies for long-insert libraries).

^c^Reads with mates mapped to the same chromosome but shows the same orientation, i.e. ‘←←’ or ‘→→’. In the case of long-insert libraries, chimera formation is one of the causes of the formation of these reads.

**Figure 1.**
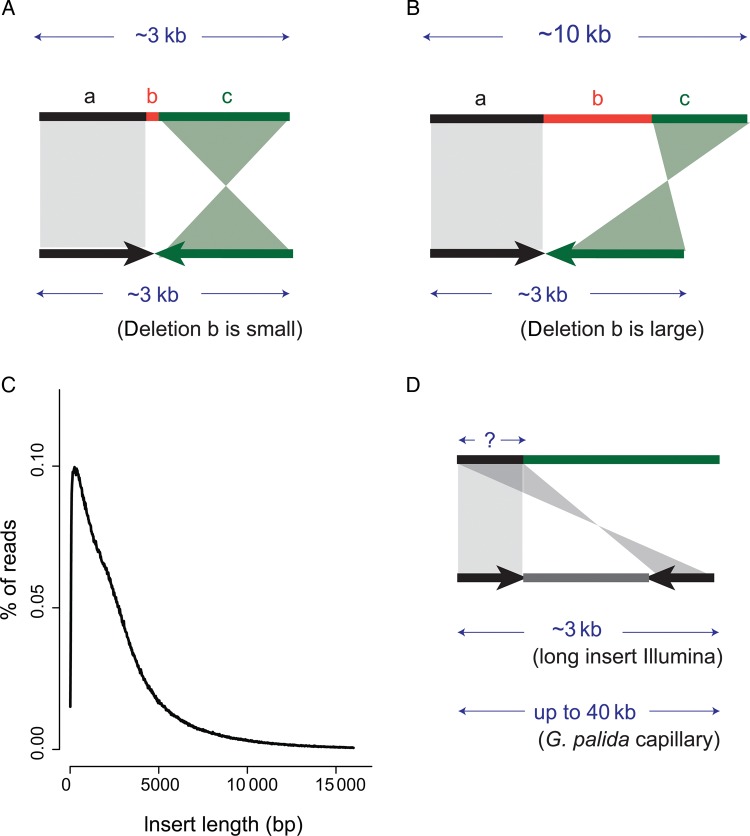
(A, B and D) Types of chimeric rearrangements. Each DNA sequence is represented by two or three adjacent segments. Arrows indicate directions of amplified fragments relative to the DNA sequence. (A) and (B) Segment a is copied, b is deleted and c is copied and reverse complemented. (D) The first part of the sequence is copied twice, with unknown sequence placed between the two copies. (C) Insert size distribution plot of wrong-orientation reads in Phi amplified libraries.

One of the main concerns in using amplification methods is their tendency to form chimeric DNA fragments, which seems to arise from a mechanism involving priming from displaced 3′ termini.^17^ Based on this mechanism, the majority of chimeric DNA fragments will be sequences where a segment, which is partially deleted, illustrated by Segments *a* and *b* in Fig. 1A, joins to another Sequence *c* from the same chromosome which is inverted. Thus we could measure the tendency of amplified libraries forming chimeras by counting the number of read pairs that were mapped with both mates in the wrong orientation (i.e. forward–forward or reverse–reverse). All amplified libraries show an increased proportion of reads in the wrong orientation when compared with the unamplified counterparts (Table 2). This pattern is more prevalent in long-insert reads, where long-insert libraries prepared using Phi and Tre show 10–12.9% of reads with the wrong orientation compared with 0.55–4.1% in short-insert libraries. The majority of these reads are evenly distributed across the chromosomes in all amplified samples (Supplementary Fig. S3), suggesting that wrongly amplified fragments occur infrequently and randomly. As mentioned before, another characteristic of the chimeras is that part of the sequence will be deleted, and as a result the two segments where they map in the genome will appear to have been brought closer together as a consequence of the deletion (Fig. 1B). Hence, when insert size is calculated based on the mapping positions, we expect to see a much broader insert size distribution than of non-chimeric reads. Indeed, this is what we observed in all WGA long-insert libraries (Replicate 1 of Phi shown in Fig. 1C and rest in Supplementary Fig. S4) with distances between mates mapped in wrong orientation sometimes even >10 kb. By further looking at sequence reads that can be uniquely mapped into two different positions on the same chromosome, we found two cases that confirm the presence of chimeras in Phi and Tre amplified fragments (Supplementary Fig. S5).

To our surprise, the insert size distribution of these chimeric reads all peaked close to 0 bp (Fig. 1C, Supplementary Fig. S4), suggesting that both mates in read pairs were overlapped or placed very close to each other. This observation cannot be solely explained by the priming mechanism where two different sequences on the chromosome were joined, and can only be explained if the same sequence is present on both ends of the sequenced fragment with one version inverted (Fig. 1D). To investigate this phenomenon further, we looked at Sanger sequence data that were produced from potato cyst nematode (*Globodera pallida)* genomic DNA, amplified using Genomiphi (Phi) and cloned into plasmid or fosmid vectors*.* Various insert sizes, from 2 kb to 40 kb (fosmid) were sequenced from either end with read lengths of 200–600 bp. Indeed, we also found that the majority of mates of wrongly oriented reads overlapped with each other, concurring that the same region was sequenced twice with one version inverted. Interestingly, we found that the fraction of wrongly oriented reads was correlated with fragment size, and in extreme cases 85% of MPs derived from fosmids mapped in the wrong orientation and overlapped each other (Supplementary Fig. S6).

### 3.3. Uniform read coverage across the genome

**Figure 2.**
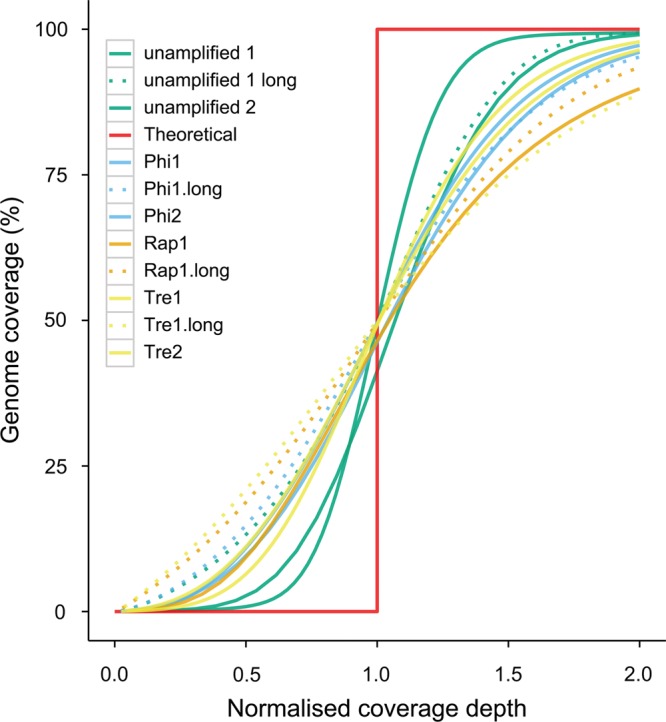
A plot of genome coverage against normalised average depth. Deviation from the theoretical curve (red) indicates less evenness in coverage depth distribution across the genome. Different protocols are plotted with different colours as listed in the legend, and dashed lines indicate read coverage from Replicate 1 of the long-insert libraries.

One of the most important criteria for accurate variant calling and assemblies from Illumina reads is an even coverage of sequence data genome-wide. We first evaluated the variability in the depth of coverage of short-insert reads^32^ by plotting the cumulative fraction of normalized depth of correctly paired read coverage that covers a given cumulative fraction of genome (Fig. 2). Normalization of read coverage depth allows libraries of different coverage depths to be compared with each other. The theoretical line (Fig. 2) indicates a perfectly uniform distribution of reads where 100% of the genome is covered by reads with a normalized and consistent depth of 1. Figure 2 shows that both replicates of the unamplified short-insert library have the closest fit to the theoretical line, suggesting the most uniform distribution of reads. The remaining samples show some level of deviation, suggesting non-uniform distribution across the genome. Distribution plots of the long-insert libraries show more deviation away from the theoretical distribution than short-insert libraries. This effect is more evident in the lower tail of the distribution, indicating a greater proportion of the genome has lower coverage. By inspecting regions of lower coverage across all libraries, the most evident patterns are regions enriched in G homopolymers tracts and GGC motifs^33^ (Supplementary Fig. S7).

**Figure 3.**
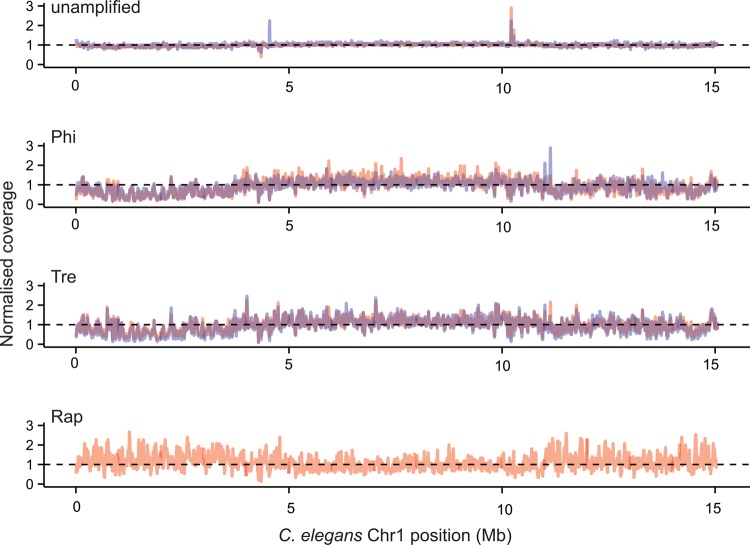
Normalized coverage of 10 kb windows on Chr 1 of *C. elegans.* Red and blue colour depicts coverage of Replicates 1 and 2, respectively.

**Figure 4.**
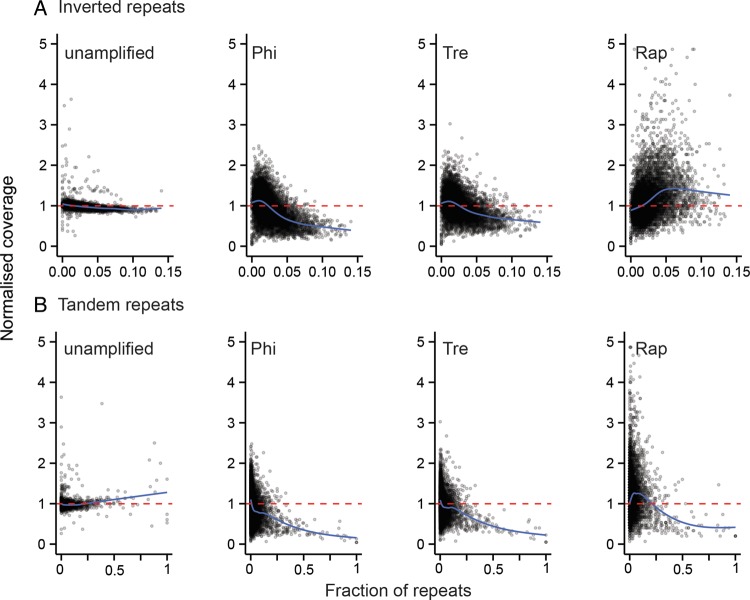
Scatterplots showing relationships between (A) inverted and (B) tandem repeat content and normalized read coverage in 10 kb windows of *C. elegans*.

Next, we grouped the Illumina short-insert read coverage at each base into 10 kb non-overlapping windows across the *C. elegans* genome. Using Chromosome I as an example, plotting the median coverage of bases in each window immediately revealed the difference in coverage between libraries (Fig. 3). Some differences exist, but two technical replicates in each library show remarkably similar patterns with each other. Libraries with unamplified DNA show more uniform coverage across Chromosome I compared with amplified libraries. Interestingly, we observed that read coverage only seems to be more even towards the middle of Chromosome I in all WGA protocols, but is even throughout in Chromosome X (Supplementary Fig. S8). The unevenness in coverage of data from Illumina short-insert libraries at *C. elegans* autosome arms mirrors the distribution of inverted and tandem repetitive sequences, which also cluster more frequently at the autosome arms^34^ (Supplementary Fig. S9). To investigate this phenomenon, we re-annotated the tandem and inverted repeat contents of *C. elegans* genome and compared them with read coverage in 10 kb windows. Strikingly, we found that both tandem and inverted repeat content are significantly correlated with read coverage across all samples including unamplified libraries (*P* < 0.001, Spearman's test). Read coverage in libraries treated with Phi and Tre decrease with increasing content of tandem and inverted repeats (Fig. 4A and B). Libraries created with Rap also show an inverse relationship between read coverage and tandem repeat content, but positively correlated with inverted repeat content, which explains contrasting patterns of coverage on autosome arms against Phi and Tre.

**Figure 5.**
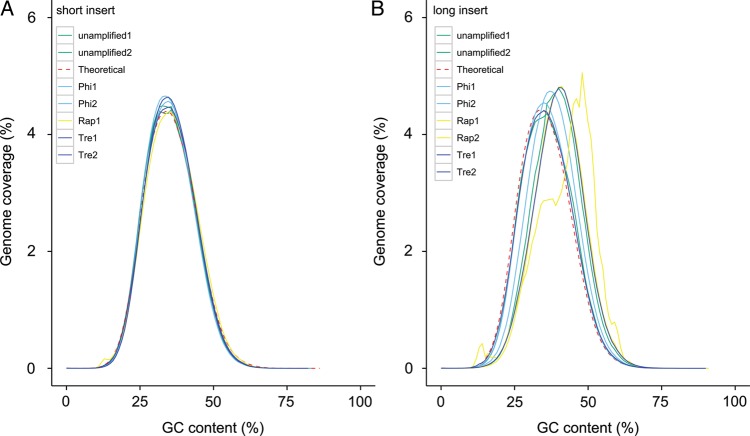
Distribution of GC content in sequenced reads of (A) short- and (B) long-insert libraries.

GC bias of Illumina reads has been widely reported in the literature.^32,35^ To analyse the effect of GC composition on uniformity of read coverage we calculated the distribution of GC of mapped reads to the reference, normalized by the average coverage across each set of replicate, and compared them against the theoretical distribution. Short-insert libraries prepared under all protocols showed a good fit to the theoretical distribution (Fig. 5A); however, we see a strong positive bias towards higher GC in all long-insert libraries (Fig. 5B). To characterize this observation further, we obtained the normalized coverage difference between short- and long-insert libraries in 10 kb windows and correlated with the GC content of the window. Compared with short-insert libraries, the majority of long-insert libraries show more amplification generated from higher extremes of GC regions and less amplification from lower extremes of GC regions (Supplementary Fig. S10).

### 3.4. Application 1: variant callings in WGA libraries

To investigate the effect on variant calling caused by the decreased uniformity in amplified libraries, SNPs were called using Varscan2^22^ on each of the short-insert replicates. Since the same starting material was used for all samples, and considering the technical variations between different sequencing runs, we first inferred 643 homozygous and 2117 heterozygous SNPs that were called in both non-amplified replicates. Only one replicate of the Rap amplification was analysed, which therefore showed the least robust SNP calling accuracy. For samples amplified with either Phi or Tre, we found ∼80% homozygous SNP calls were also called in at least one replicate (Table 3A). The majority of miss calls in libraries prepared using these two protocols from sites with a coverage depth ≤2, consistent with the expectation that some regions of the genome failed to amplify during the process. The effect of non-uniform read coverage of amplified libraries is further reflected in their heterozygous calls, where only 61–65% of SNPs from the non-amplified library were also called in at least one of the replicates from the two amplification protocols. An exhaustive investigation of the miss-called SNPs revealed that most of the missed heterozygotes were incorrectly called as homozygous (Table 3B).

**Table 3.** Summary of variant calls

Protocols	Phi	Tre	Rap
(A)			
Homozygous SNPs (643)			
No/low coverage	105	91	192
Not called the same	28	30	28
Also called in one replicate	150	154	423
Called in both replicates	360	368	NA
Heterozygous SNPs (2117)			
No/low coverage	132	85	291
Not called the same	692	650	832
Also called in one replicate	705	813	994
Called in both replicates	588	569	NA
(B)			
Homozygous SNPs			
Called differently in both unamplified replicates	37	36	107
Called in one replicate	14	28	134
Heterozygous SNPs			
Called differently in both unamplified replicates	105	158	528
Called in one replicate	44	46	1465

(A) Fate of 643 homozygous and 2117 heterozygous SNP calls from both unamplified replicates; (B) fate of additional homozygous and heterozygous SNP calls from amplified replicates.

### 3.5. Application 2: assembly generated by WGA prepared libraries

In order to evaluate the effect that different WGA protocols may have in generating a genome assembly, we used the SGA assembler^28^ to assemble reads from each of the libraries. These assemblies were then assessed using GAGE^30^ and summarized in Table 4**.** We only presented Replicate 1 for this analysis because of the much lower coverage in Replicate 2 observed in all libraries, with the smaller numbers of reads producing significantly worse assemblies (Supplementary Table S2). Having a genome reference allowed us to compute corrected N50 where contigs were broken at miss-assembled regions prior to the calculation of N50. The first observation is that the Rap amplified libraries produce the worst assembly, and as expected the most contiguous and accurate assembly is produced from unamplified library. The second observation is that the assemblies produced from Phi and Tre amplified libraries are almost identical to unamplified libraries, with only an additional 0–4.3% of the assembly missing compared with the reference and similar corrected N50 values. The variation in assembly quality between replicates was greater than between non-amplified and amplified libraries prepared by following either Phi or Tre protocols (Supplementary Table S2).

**Table 4.** Summary statistics of assembly and scaffolding data from different libraries

Protocol	Contig assembly				Scaffolding			
	Unamplified	Phi	Tre	Rap	Unamplified	Phi	Tre	Rap
Assembly size (bp)	94 641 187	94 028 877	94 541 978	88 411 985	96 571 590	96 849 620	96 382 191	96 869 541
contig number	13 386	14 661	21 721	34 073	9415	8744	9068	9247
contig average (kb)	7.1	6.4	4.4	2.6	10.3	11.1	10.6	10.5
largest contig (kb)	167.7	147.8	116.8	41.8	167.7	187.5	167.7	167.7
N50 (kb)	16.6	15.7	10.8	4.2	17.6	24.1	20.7	18.4
N50 (number)	1525	1597	2109	5897	1533	1067	1258	1482
GAGE assessment								
Corrected N50 (kb)	15.1	14.1	9.5	3.5	16.8	22.7	19.7	17.7
Corrected N50 (number)	1721	1825	2431	7493	1642	1141	1354	1577
Missing reference (%)	0.09	0.09	4.43	0.14	0.09	0.09	0.09	0.09
Inversion	13	21	38	50	12 (−1)	17 (+4)	13	15 (+2)
Relocation	7	7	11	22	17 (+10)	13 (+6)	19 (+12)	11 (+4)
Translocation	12	16	37	30	12	12	12	11 (−1)

To assess the effect of the uneven sequencing coverage and the presence of erroneous orientation in the long-insert libraries, we ran the SSPACE^29^ scaffolder on the best assembly (non-amplified Replicate 1) using reads from each long-insert library. We only used Replicate 1 for this analysis because of the much lower coverage in Replicate 2 observed in all samples. A total of 10 additional miss-assemblies were identified by GAGE after scaffolding with the unamplified long-insert library (Table 4) and, comparably, the Phi amplified library created the same number of miss-assemblies but actually had the largest corrected N50, as well as containing the largest scaffold of all the assemblies. The assembly scaffolded with the Rap amplified library, which contains the fewest wrongly oriented reads in among all amplified libraries but has the most PCR duplicates, shows the fewest additional miss-assemblies and with both the N50 and longest scaffold length very similar to the unamplified sample.

## 4. Discussion

### 4.1. Bias due to WGA protocols and genome features

There are two main types of biases present in Illumina read sequenced from amplified DNA: those that arise from the Illumina library construction and sequencing process, and those arising from the nature of DNA fragments generated from WGA. Reads of different insert size also exert influences on the extent of these biases. First, inverted repeats are known to generate chimeras in WGA protocols,^17^ the extent of which we assessed by recording the number of wrongly oriented reads in the samples. In fact, the distribution of wrongly oriented reads reveals that previously proposed mechanism^17^ only constitute a part of these, and instead the majority of these reads arise from fragments containing the same sequence on both ends with one end being an inverted version of another. The same trend was found in the *G. pallida* capillary reads, and a positive correlation was observed between numbers of chimeras and length of sequenced fragments. An explanation could be that the duplicated sequences are more likely to be present in larger fragments. Hence selection of such fragments in larger insert libraries after shearing will also result in preferentially selection for artefacts. In shorter insert Illumina libraries, the wrongly amplified sequences were broken down into smaller fragments and hence a decreased number of wrongly orientated reads was found. It would be interesting to sequence the whole wrongly amplified fragment to reveal the dominant nature of chimera formation in WGA.

Secondly, we obtained the quality statistics of read libraries such as read coverage aligned across the genome, proportions of wrongly oriented mapped reads and searched for potential biases due to GC content, repeat composition of the *C. elegans* genome and the different insert size libraries. Coverage biases in regions of genomes as a result of amplification have been previously observed in bacteria^16^ and in humans,^15^ and we found that the uneven coverage between chromosome arms and centres are correlated with tandem and inverted repeat content, both of which have been previously described to bias the coverage of WGA reads.^16,17^ We show that in repeat regions Phi and Tre displayed lower read coverage, while Rap showed different biases depending on the repeat type, suggesting alternative mechanisms affecting the amplification process. It also may not be the repeat sequence *per se*, as repetitive regions in the genome are also associated with the global structural features such as histone modifications. For example, chromosomal arms of *C. elegans* show enrichment of H3K9 methylation,^36^ which are also zones of elevated meiotic recombination.^34^

Thirdly, we found the presence of GC bias in the reads from long-insert libraries including those without WGA, despite the *C. elegans* genome possessing an almost uniform GC content (36%) across all chromosomes.^34^ The current proposed mechanisms behind WGA do not explain a bias with GC content, and indeed we found no obvious effect of WGA libraries against GC content. Hence, the overall bias is mainly caused during the library construction process of the long-insert libraries and will be even more prevalent in genomes that possess more extreme GC content, such as *Plasmodium falciparum* (17% GC).

Finally, in this study we have analysed a total of three WGA protocols. We found that the addition of trehalose (Tre) does not seem to improve the coverage evenness against the libraries amplified with *Genomiphi* only (Phi), as all the metrics and biases are almost identical between two protocols. A possible explanation is that *Genomiphi* might have already contained reagents that work similarly to trehalose. On the other hand, libraries amplified using Rapisome (Rap) showed different patterns to *Genomiphi* based protocols. Constructing a good Illumina library from Rap amplified products seemed to be more difficult than Genomiphi based protocols, considering the lower number of mapped reads and higher number of PCR duplicates. However, Rap amplified libraries have shown lower number of wrongly orientated reads, suggesting an alternative mechanism that attributes to biases to different genome features. For instance, we show regions associated with inverted repeat actually increase read coverage in Rapisome libraries. It will be of interest to find out whether the lower percentage of wrongly orientated reads in Rapisome amplified libraries are also noticeable in longer insert libraries. If that was indeed the case, then Illumina libraries can be constructed from much larger amplified DNA fragments with still lower proportions of wrongly oriented reads.

### 4.2. Performance of WGA reads

Sequence reads from amplified fragments can potentially give rise to false SNP calls, particularly in genomic regions that are difficult to amplify. We found that SNPs were under-called from reads generated from amplified DNA compared with unamplified samples. When reads were sequenced at good coverage, we found that ∼80% of homozygous SNP candidates from unamplified samples could also be identified correctly from amplified samples, while the majority of miss calls were due to insufficient read coverage. The limitation seems to be more prevalent in heterozygous SNPs, where only up to 65% of candidates could be accurately identified. Most of these SNPs were called as homozygous in one of the alleles, and this may be due to the fact that most SNP callers make heterozygous calls confidently if allele frequencies were close to 50%, for which non-uniform coverage results in departure from this assumption. Hence, although most of the homozygous SNPs can be accurately identified in WGA samples, it is essential to investigate the allele frequencies of variant sites in order to not miss calls that were otherwise heterozygous in the sample.

There have been advances in assembly algorithms that assemble genomes with fluctuating coverage in the samples, but we wished to emphasize the effect that reads sequenced from amplified fragments have on a typical assembly process, hence we applied commonly used methods to assemble the *C. elegans* genome using short-insert reads with the SGA assembler,^28^ and scaffolded using long-insert reads with the SSPACE scaffolder.^29^ Contig and scaffold statistics generated from amplified libraries were comparable with those from their unamplified counterparts. In fact, we found that variability between technical replicates influenced assembly metrics more than different WGA protocols. We also found that the wrongly oriented reads did not cause more miss-assemblies than expected. An explanation is that our long-insert libraries were sequenced at adequate coverage and problematic reads were present in minority. Occasions can arise where correct reads suggest joining of two scaffolds but problematic reads suggest otherwise, in which case the SSPACE scaffolder will scaffold correctly because the number of correct reads outweighs problematic reads. However, we expect that reads from larger insert WGA libraries would result in significantly more scaffolding errors because the majority of reads are problematic, with the error rate increasing with fragment length.

### 4.3. Conclusion

WGA will remain an essential method in genomics for many years to come. The mechanisms of chimera formation and other sources of bias have been investigated and there are many new protocols that are currently being developed to minimize such effects. In the meantime, it is also important to evaluate the nature of biases in order to correctly interpret assembly results. Here, we have presented the effects of WGA on Illumina reads. We recognize the biases present from the reads, and show that the DNA amplified from all protocols investigated in this article is able to call the majority of SNPs and produce accurate assemblies comparable with those produced from unamplified DNA. Reads sequenced from longer fragments are still useful in scaffolding and problematic reads can be detected and filtered out as long as they remain a minority. We envisage also using WGA in other eukaryotic species (including nematodes) that possess similar GC content and base composition to *C. elegans*. Fundamentally, as biologists turn to sequencing more unexplored species or samples from which only very low amounts of starting material are available, our study provides a first initial assessment on Illumina sequencing from WGA DNA.

## 5. Availability

Illumina data are released to the European Nucleotide Archive (http://www.ebi.ac.uk/ena/) under accession number ERP000964.

## Supplementary data

Supplementary data are available at www.dnaresearch.oxfordjournals.org.

## Funding

This work was supported by JSPS KAKENHI (grants numbers 20353659 and 23248024) and the Wellcome Trust (grant no. WT 098051). I.J.T. was supported by JSPS Postdoctoral Fellowship Program for Foreign Researchers.

## Supplementary Material

Supplementary Data
