# Supplementary material for: Summarizing Specific Profiles in Illumina Sequencing from Whole-Genome Amplified DNA
Source: DNA Res. 2013 Dec 18;21(3):243–54. doi: 10.1093/dnares/dst054 (PMC4060946; doi:10.1093/dnares/dst054)
Supplement: Supplementary Data [file supp_21_3_243__index.html]

Summarizing Specific Profiles in Illumina Sequencing from Whole-Genome Amplified DNA — Summarizing Specific Profiles in Illumina Sequencing from Whole-Genome Amplified DNA — Supplementary Data 

# Summarizing Specific Profiles in Illumina Sequencing from Whole-Genome Amplified DNA

## Supplementary Data

Supplementary Data

**Files in this Data Supplement:**

- Supplementary Data - Docx file
