# Supplementary material for: Summarizing Specific Profiles in Illumina Sequencing from Whole-Genome Amplified DNA
Source: DNA Res. 2013 Dec 18;21(3):243–54. doi: 10.1093/dnares/dst054 (PMC4060946; doi:10.1093/dnares/dst054)
Supplement: Supplementary Data [file supp_dst054_dst054supp.docx]

SUPPLEMENTARY FIGURES AND TABLES

Supplementary Figure S1 – Whole genome amplification (WGA) experimental setup and construction of Illumina short and long libraries. Red rectangles indicate stages where problematic reads arise in the library construction process.


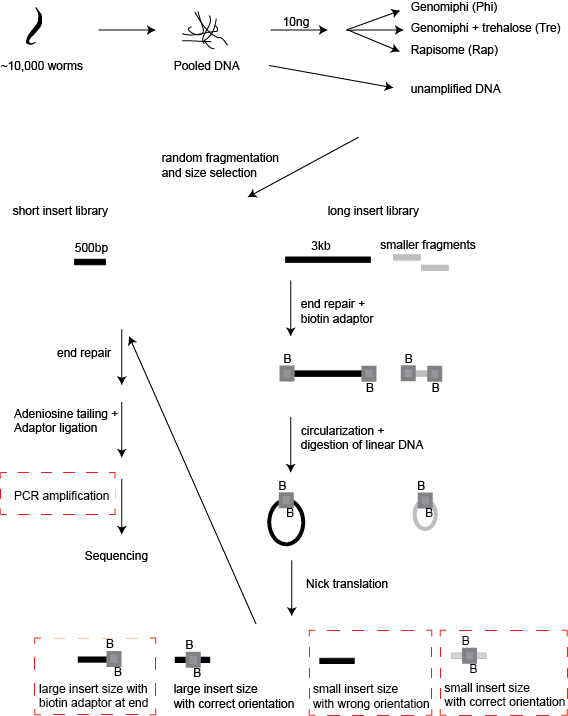


Supplementary Figure S2 – Insert size distribution of correct orientation reads from different protocols. Dashed and solid lines indicate distributions from unamplified and three different amplified libraries, respectively. Additional illustration from Supplementary Figure S1 is drawn on replicate 2 of the long insert library to explain bimodal peaks.


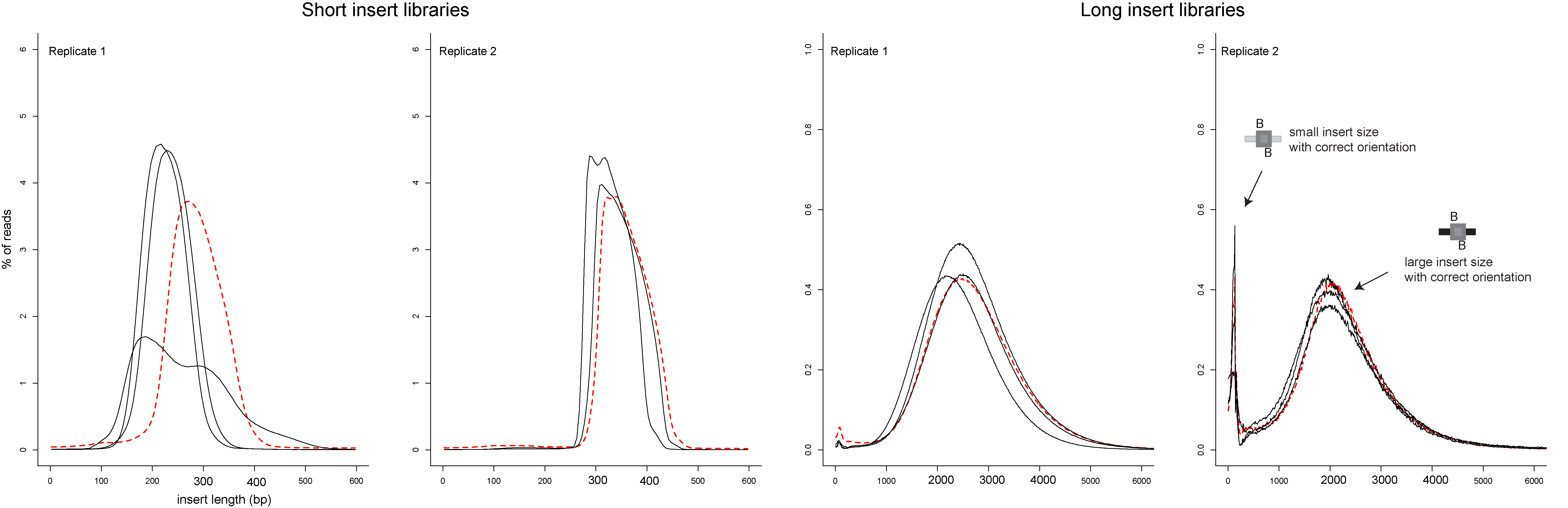


Supplementary Figure S3 – Coverage of wrong-orientation reads in replicate 1 of long insert libraries along the *C. elegans* genome.


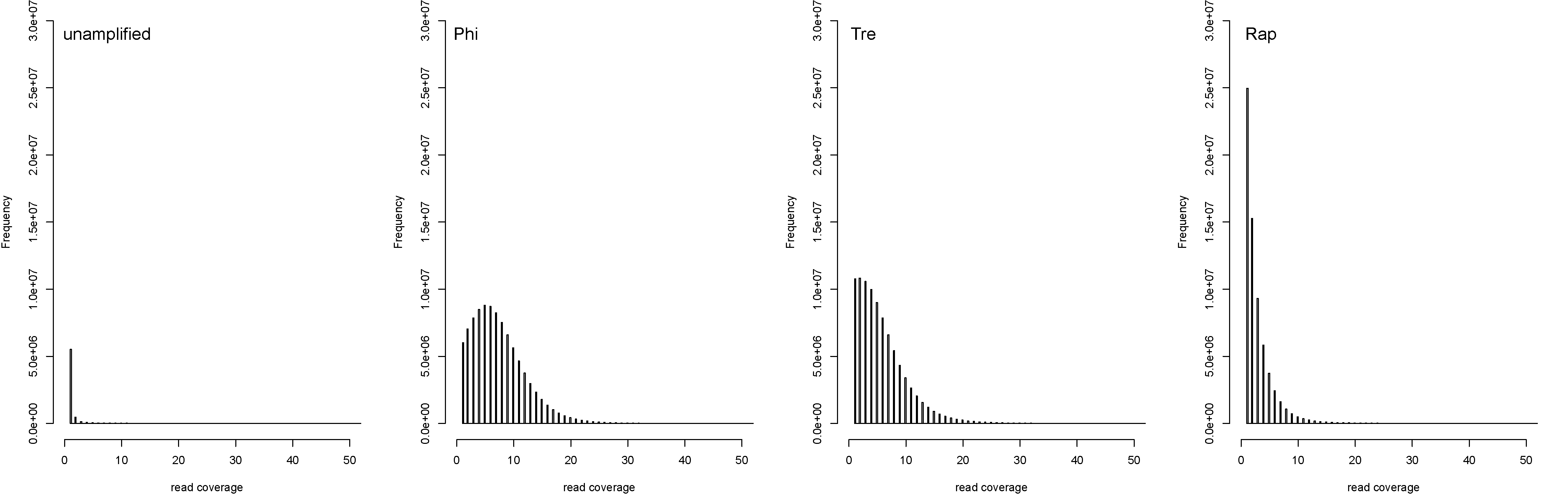


Supplementary Figure S4 – Distribution of wrong-orientation reads in long insert libraries. Black and blue lines indicate distributions of replicate 1 and 2, respectively.


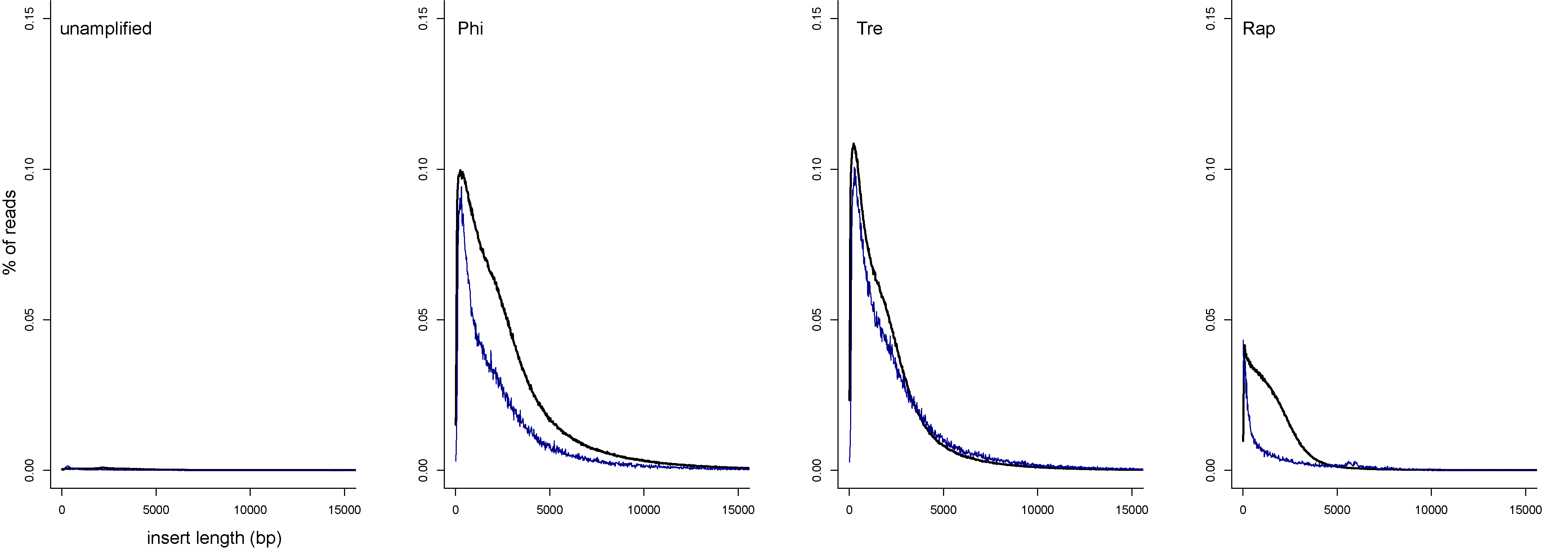


Supplementary Figure S5 – Possible scenarios of split sequence reads that are suggestive of chimera formation in amplified fragments. R and r represent two possible scenarios if a mate of the read pair contains the chimera junction. A total of two cases were found indicating chimera formation in amplified fragments.


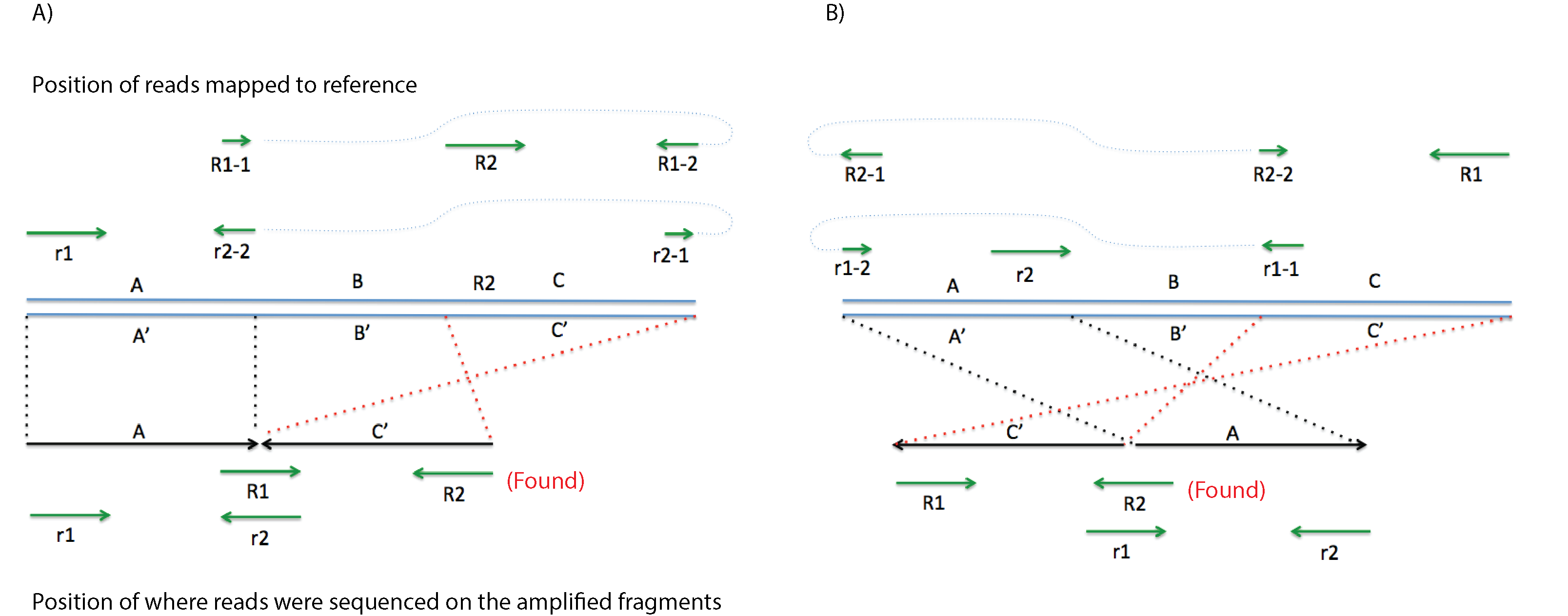


Supplementary Figure S6- Insert size distribution of correct orientation (black) and wrong-orientation capillary reads (red) of potato cyst nematode *G. pallida.*


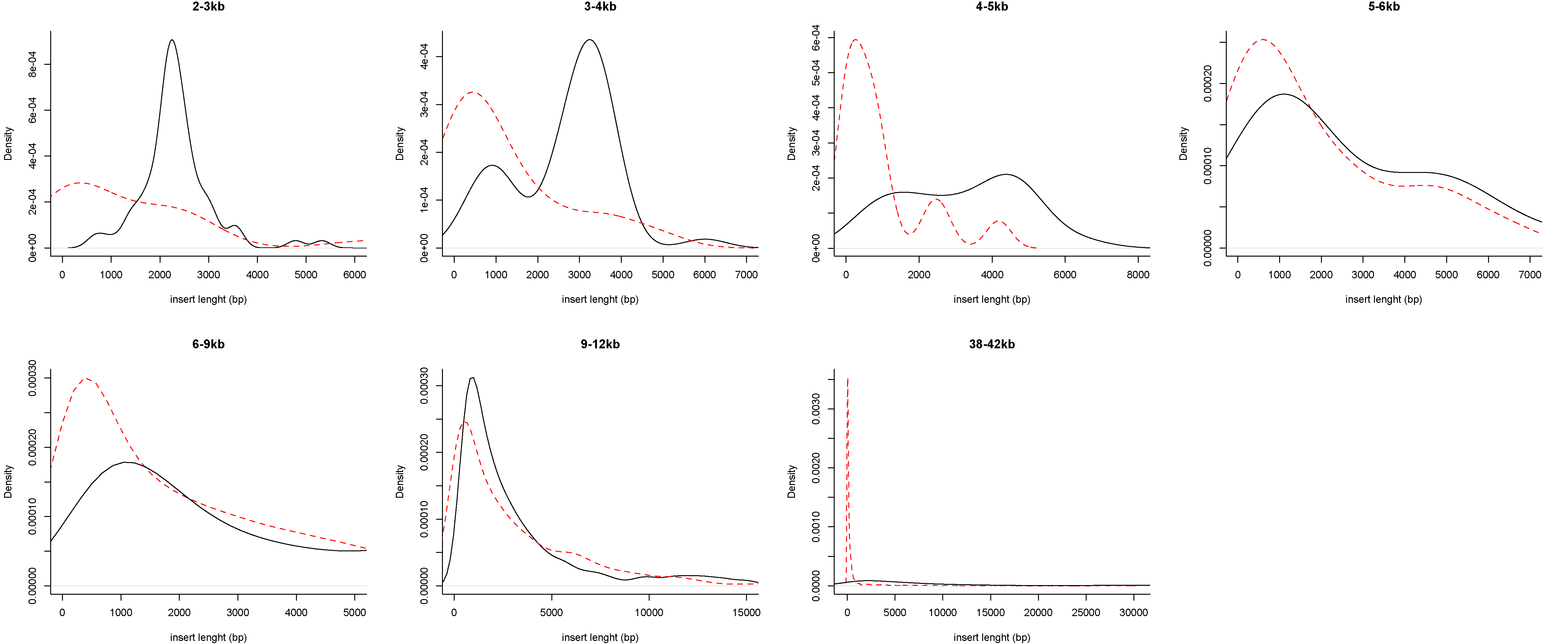


Supplementary Figure S7 – A typical low coverage region in *C. elegans*. Colour lines shows the coverage of sequence read depth in replicate one of short insert libraries across all methods.


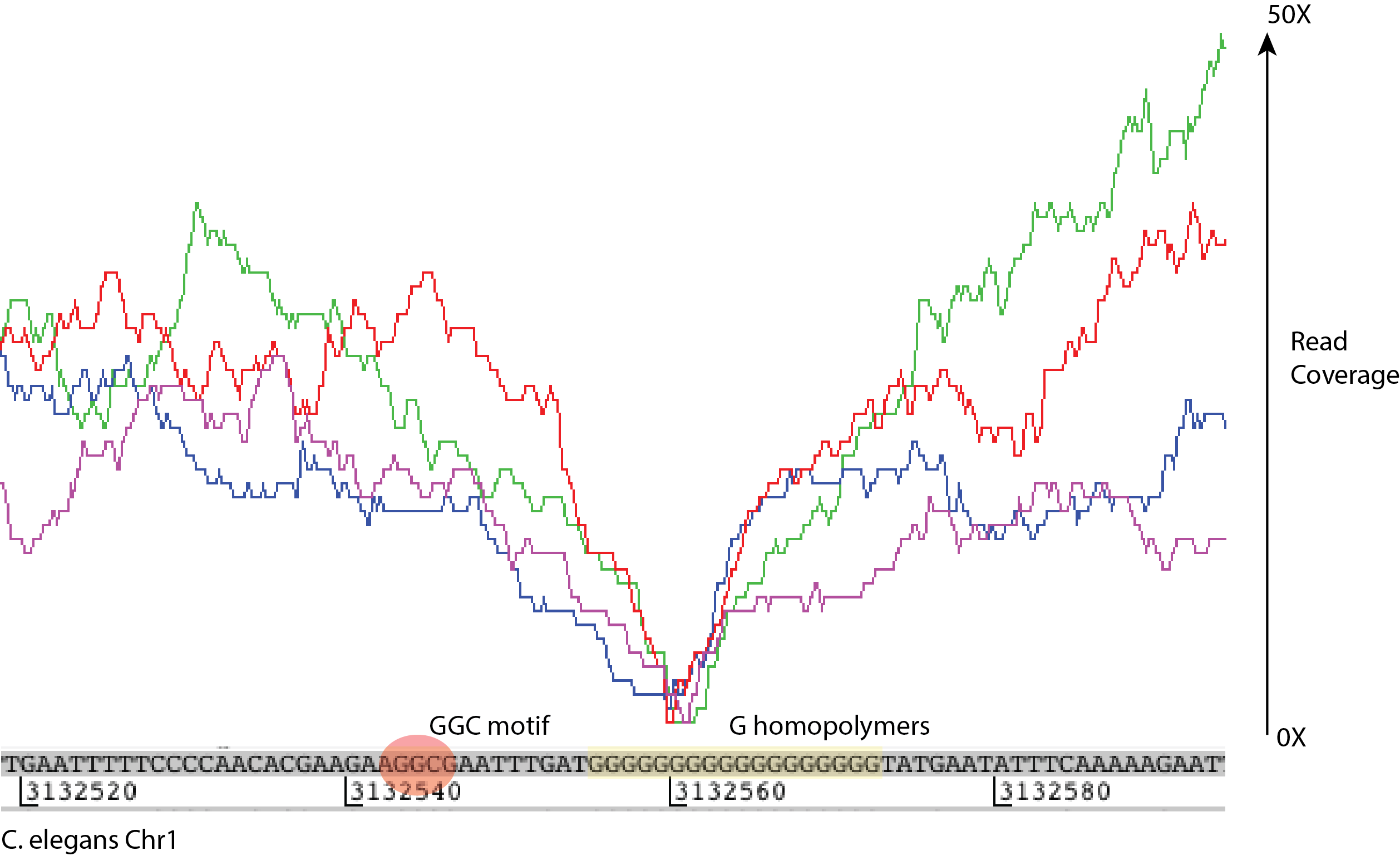


Supplementary Figure S8 – *C. elegans* Chr X 10kb window normalised sequence read coverage.


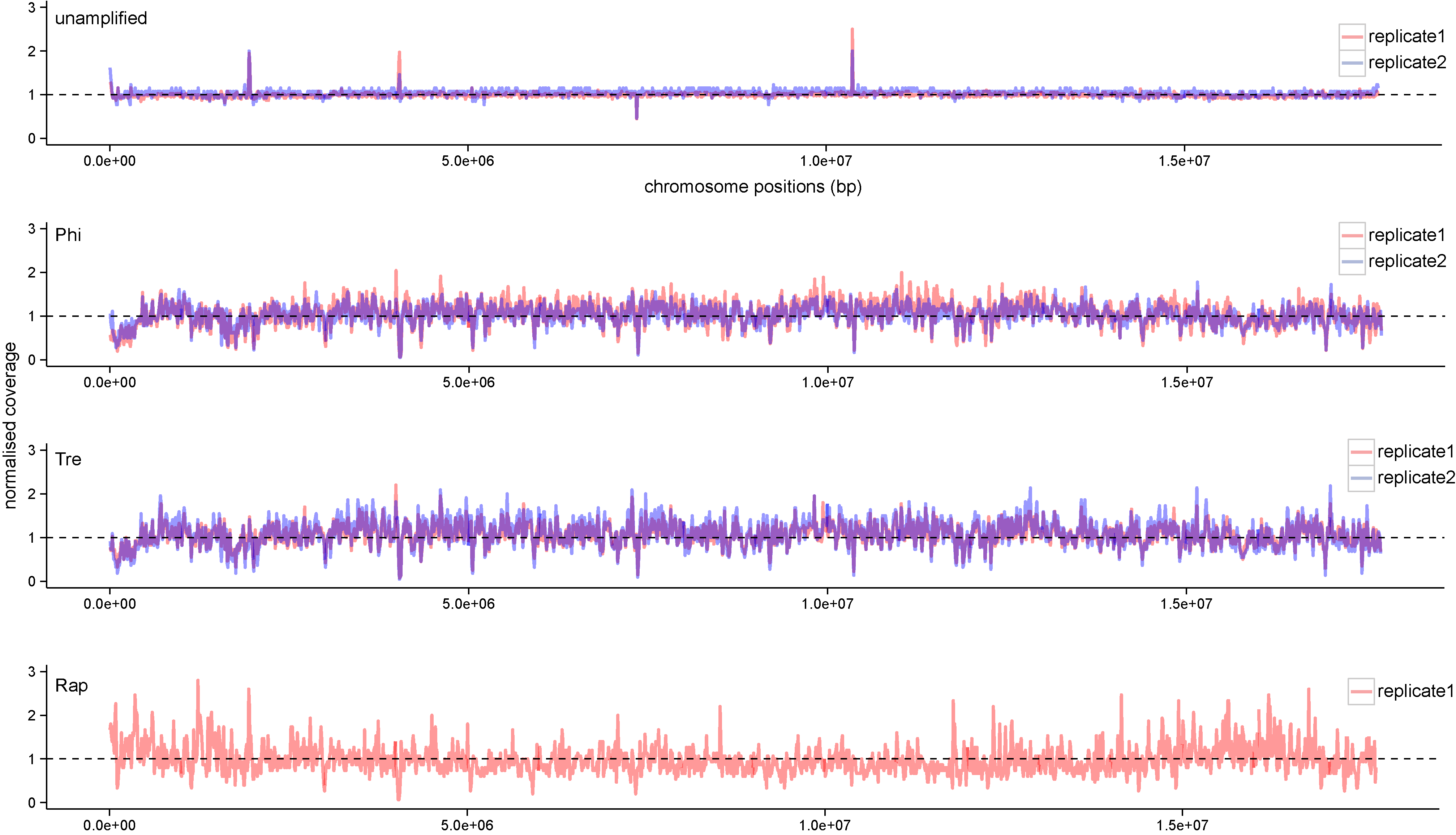


Supplementary Figure S9 - *C. elegans* Chr1 normalised coverage, tandem and inverted repeat content in 10kb windows.


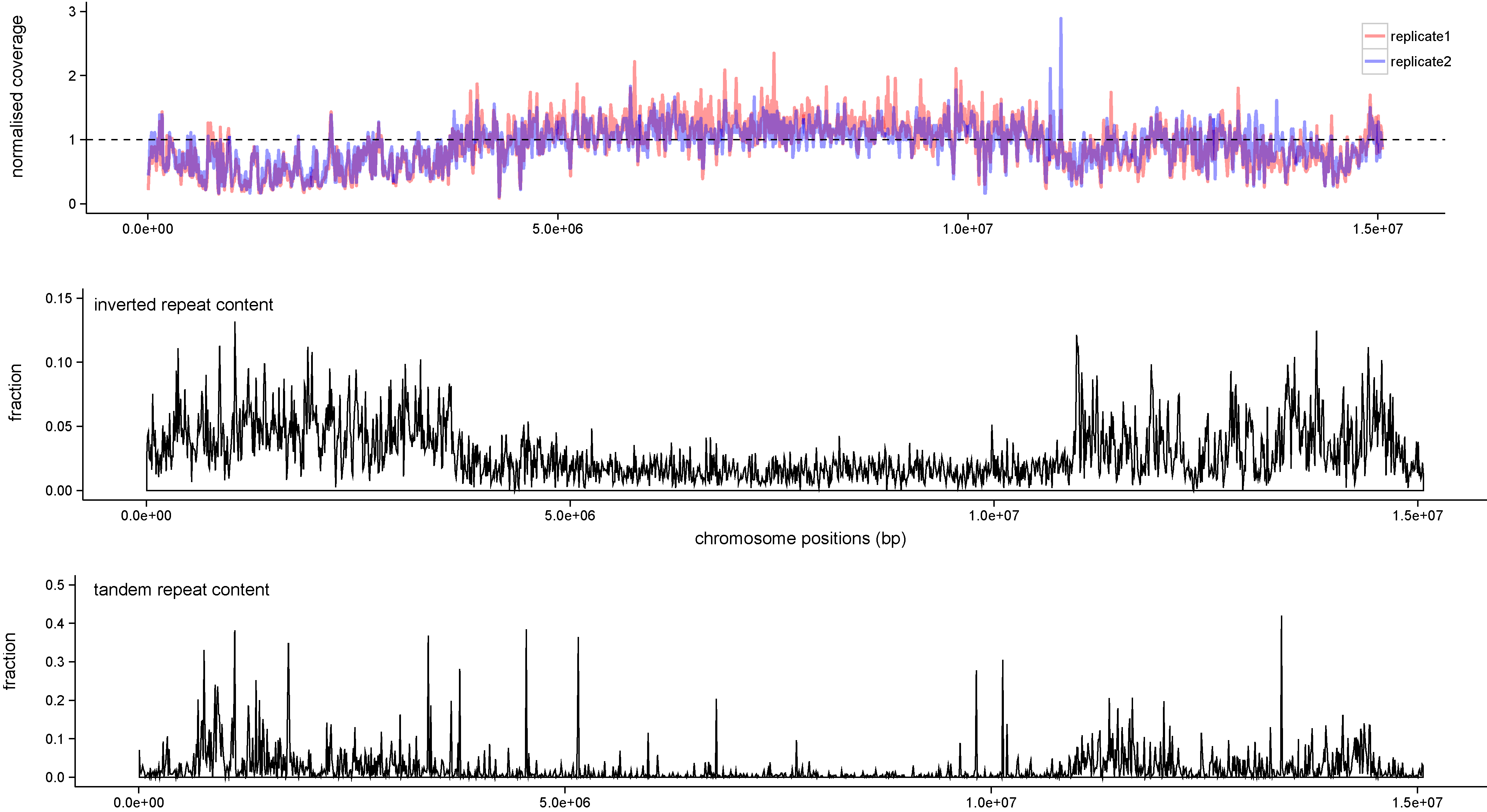


Supplementary Figure S10- Relationship of read coverage difference between short and long insert libraries against GC content in 10kb windows of *C. elegans* genome.


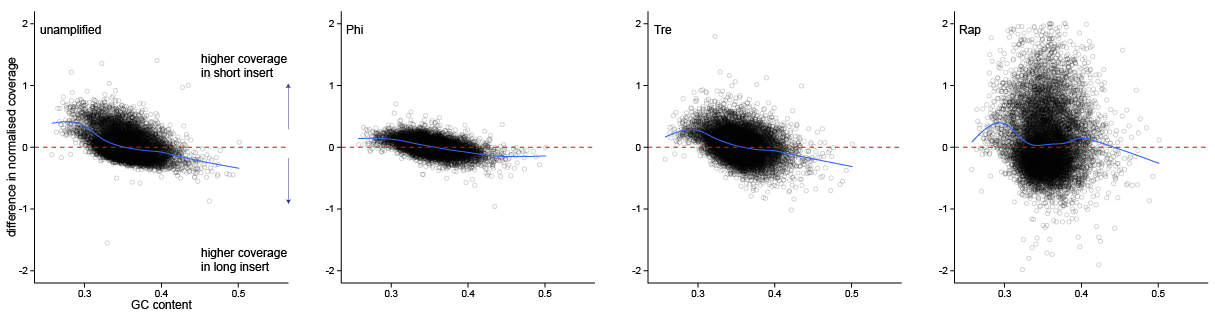


Supplementary Table S1 – Summary statistics of read trimming prior to mapping to *C. elegans* genome.

| Protocols | Replicate | Insert | Input Read Pairs | Both Surviving | Forward Only Surviving | Reverse Only Surviving | Dropped |
| --- | --- | --- | --- | --- | --- | --- | --- |
| unamplified | 1 | short | 21436561 | 20844338 (97.24%) | 434061 (2.02%) | 122345 (0.57%) | 35817 (0.17%) |
| Phi | 1 | short | 24713632 | 24250958 (98.13%) | 300641 (1.22%) | 132097 (0.53%) | 29936 (0.12%) |
| Rap | 1 | short | 14654510 | 13138699 (89.66%) | 360948 (2.46%) | 992415 (6.77%) | 162448 (1.11%) |
| Tre | 1 | short | 22696287 | 22240635 (97.99%) | 308471 (1.36%) | 118617 (0.52%) | 28564 (0.13%) |
| unamplified | 2 | short | 8358641 | 8166866 (97.71%) | 156446 (1.87%) | 25738 (0.31%) | 9591 (0.11%) |
| Phi | 2 | short | 10555135 | 10328148 (97.85%) | 168577 (1.60%) | 42261 (0.40%) | 16149 (0.15%) |
| Rap | 2 | short | 11480864 | 11139067 (97.02%) | 223124 (1.94%) | 105527 (0.92%) | 13146 (0.11%) |
| Tre | 2 | short | 12826863 | 12594394 (98.19%) | 181397 (1.41%) | 39197 (0.31%) | 11875 (0.09%) |
| unamplified | 1 | long | 33637595 | 30428430 (90.46%) | 1624730 (4.83%) | 1527466 (4.54%) | 56969 (0.17%) |
| Phi | 1 | long | 33457445 | 30867605 (92.26%) | 1319211 (3.94%) | 1239987 (3.71%) | 30642 (0.09%) |
| Rap | 1 | long | 33,456,703 | 29221828 (87.34%) | 2116953 (6.33%) | 2008753 (6%) | 109169 (0.33%) |
| Tre | 1 | long | 32946396 | 27921293 (84.75%) | 2524947 (7.66%) | 2379198 (7.22%) | 120958 (0.37%) |
| unamplified | 2 | long | 1443372 | 1275860 (88.39%) | 89200 (6.18%) | 77990 (5.4%) | 322 (0.02%) |
| Phi | 2 | long | 1622160 | 1380288 (85.09%) | 123844 (7.63%) | 117306 (7.23%) | 722 (0.04%) |
| Rap | 2 | long | 2096250 | 1957311 (93.37%) | 73358 (3.5%) | 65351 (3.12%) | 230 (0.01%) |
| Tre | 2 | long | 1680455 | 1499928 (89.26%) | 93535 (5.57%) | 86696 (5.16%) | 296 (0.02%) |

Supplementary Table S2 – Summary statistics of assembly data from different libraries with each library down-sampled to have the same number of reads as the lowest library (replicate 2 of unamplified library).

| Protocol | unamplified | | Phi | | Tre | | Rap |
| --- | --- | --- | --- | --- | --- | --- | --- |
| Replicate | 1 | 2 | 1 | 2 | 1 | 2 | 1 |
| Assembly size (bp) | 92,999,424 | 94,665,381 | 83,593,606 | 86,061,997 | 86,686,207 | 85,327,395 | 66,889,869 |
| contig number | 31,932 | 41,964 | 29,139 | 35,239 | 32,159 | 33,195 | 50,401 |
| contig average (kb) | 2.9 | 2.3 | 2.9 | 2.4 | 2.7 | 2.6 | 1.3 |
| largest contig (kb) | 37.7 | 167.7 | 89.2 | 48.1 | 54.6 | 66.8 | 20.6 |
| N50 (kb) | 4.4 | 3.1 | 6.2 | 4.2 | 5.1 | 4.9 | 1.6 |
| N50 (num) | 6,239 | 8,465 | 3,272 | 5,245 | 4,217 | 4,069 | 11,631 |
| **GAGE assessment** |  |  |  |  |  |  |  |
| Corrected N50 (kb) | 4.0 | 2.6 | 4.3 | 3.2 | 3.9 | 3.5 | 0.9 |
| Corrected N50 (num) | 7,131 | 10,885 | 4,925 | 7,256 | 5,773 | 5,903 | 25,815 |
| Missing Reference (%) | 0.08 | 4.87 | 0.06 | 3.59 | 0.06 | 3.34 | 0.16 |
| inversion | 45 | 45 | 58 | 64 | 69 | 58 | 84 |
| relocation | 17 | 34 | 37 | 39 | 38 | 35 | 49 |
| translocation | 48 | 65 | 54 | 54 | 65 | 55 | 85 |
